# Progress in Medicine

**Published:** 1898-04-02

**Authors:** 


### Progress in Medicine.

### DISEASES OF THE DIGESTIVE ORGANS.

(*Continued from p. 451, Vol. XXIII.*)

**Cholelithiasis.**—W. Hunter[16] refers to the great prevalence of gall-stones, and estimates that 7 per cent. of the population suffer from them. Their causation is very obscure, and depends not on any general constitutional disturbance, but on some local disorder of the mucous membrane of the bile passages, followed by an increase of the cholesterin normally present, and its deposition round any particle of insipissated bile which may be present. A large proportion of the stones consist of bilirubin-calcium alone, but very many have cholesterin deposited round a nucleus of that material. Those formed low down—that is in the gall-bladder—are chiefly composed of cholesterin, which appears to be a derivative of degenerated epithelium. Even the bilirubin and calcium do not normally combine, except where some solution of albumen is present. Hence a catarrhal state of the epithelium of the ducts seems to be the cause of the precipitation of all the constituents of gall-stones. This catarrh is set up not by stagnation only, but by that combined with infection, either, as a rule, from the B. coli or typhosus, or from one of the pyogenic organisms. Excretion of certain poisons from the liver in dyspepsia and heart failure may also set up catarrh in the intra-hepatic ducts. As to treatment, active exercise, the use of salicylate of soda, and the avoidance of tight lacing are important means towards preventing stagnation and the production of calculi. In the discussion on this paper, J. E. Graham referred to the value of the discovery of minute quantities of bile pigment in the urine when there is a difficulty in the diagnosis of cholelithiasis, and gave an exhaustive account of the symptoms and results of the various forms of obstruction produced by gall-stones. Osler spoke of the importance of typhoid fever in causing the formation of gall-stones, and noticed the curious fact that the typhoid bacillus, like the pneumococcus, can exist in the body without producing typhoid fever.

A remarkable proof of the length of time jaundice from the impaction of a stone may continue is afforded by a case reported by Dr. Brockbank. The patient showed the ordinary symptoms of biliary obstruction, with jaundice, which continued without interruption until her death from acute phthisis, twenty months after it first appeared. At the post-mortem, a solitary gall-stone was found fixed in the common duct; and, from the appearance of the stone, the writer was led to believe that no others had existed, and that this one had probably been formed after an attack of typhoid 18 years before. Another remarkable case, a spindle-celled sarcoma of the gall-bladder with calculi, is reported by Griffon and Segall.[17]

Kelynack[18] gives a valuable review of the facts known up to the present time as to growths occurring in the gall-bladder and ducts. It appears that while malignant growths of the gall-bladder are found much oftener in women than in men, just as gall-stones are met with in them four or five times as frequently as in men; on the other hand, growths of the bile ducts are found as often in men as in women. The most common age is between 50 and 60, or rather later than that when carcinoma develops elsewhere. An important fact is the frequency with which gall-stones are present with growths of the gall-bladder. While gall-stones are found in from 6 to 12 per cent. of all post-mortems, they appear in from 90 to 100 per cent. of those where these malignant growths exist. It has been argued that the irritation they cause favours the development of the growths, and on the other hand that the growths give rise to conditions favourable to the formation of calculi. However, as against the latter theory, it may be urged that gall-stones are not frequently found where secondary cancerous growths of the gall-bladder exist. The relations of cancer affecting the ducts to gall-stones is not so clear. The type of growth is usually that of carcinoma; the most common is the columnar-celled, but the place of origin is not easy to determine. When in the ducts the growth often arises near the termination of the common bile duct. The growth may be nodular, diffuse, or occasionally villous; and secondary deposits are found most often in the liver, and sometimes in the peritoneum and adjacent organs. The symptoms include the presence of a tumour in more than half the cases, which is usually firm and often moveable. Jaundice may or not accompany a growth in the bladder, and is transitory, persistent, or recurring, but when the ducts are involved it is found universally. Pain is very variable, and vomiting or derangement of the bowels may be present, as well as pyrexia, wasting, and cholæmia. On the whole growths in the ducts cause more jaundice and cholæmia with more complete absence of bile from the intestines, and seldom produce an appreciable tumour. Cholelithiasis is difficult to distinguish, especially as it may be present together with a growth, but in it the jaundice is often less intense and persistent; there may have been attacks of colic, and the gall-bladder is rarely distended. Still, these signs cannot altogether be relied on for diagnosis, and there are a large number of affections of neighbouring organs, which are also difficult to distinguish. Early surgical exploration and removal can alone cope with these growths, and, in view of their possible origin from the irritation of gall-stones, we have a good reason for such a course where the disease may possibly be only cholelithiasis.

**Acute Yellow Atrophy**[19] has been the subject of much study, one of the most recent points made out about it being the fact that, as in other diseases produced by a general intoxication, we may have changes produced in the spinal cord. Goldscheider and Moxter report changes corresponding very nearly to those found in fatal anæmias. The neuroglia elements were increased in size, but not in number. There were here and there swollen axis cylinders and patches of degeneration in the parenchyma. The precise nature of the poison remains in doubt, but Von Kahlden notices[20] the number of cases which occur after infectious diseases and syphilis, as well as the influence of arsenic and phosphorus. There appears to be also some connection between acute yellow atrophy and the bacterium coli. Von Kahlden goes on to consider the results in those cases which are not fatal, and of which he has collected a certain number of instances. From autopsies on cases of his own and those reported elsewhere he argues that where the disease does not rapidly terminate in death, the liver soon shows both the macroscopical and minute appearances of cirrhosis; and, in fact, typical cases of cirrhosis develop from these acute attacks of yellow atrophy. He particularly refers to the history of a patient, who, after osteomyelitis and a staphylococcal infection was the subject of yellow atrophy, and in whom cirrhosis of the liver was found post-mortem, produced, as he thinks, by the acute attack.

